# Carbon monoxide releasing molecule-2 (CORM-2) inhibits growth of multidrug-resistant uropathogenic *Escherichia coli* in biofilm and following host cell colonization

**DOI:** 10.1186/s12866-016-0678-7

**Published:** 2016-04-12

**Authors:** Charlotte Sahlberg Bang, Robert Kruse, Kjell Johansson, Katarina Persson

**Affiliations:** Faculty of Medicine and Health, Örebro University, SE-701 82 Örebro, Sweden; iRiSC - Inflammatory Responses and Infection Susceptibility Centre, Örebro University, SE-701 82 Örebro, Sweden

**Keywords:** Carbon monoxide releasing molecule, CORM-2, Extended-spectrum β-lactamase, Uropathogenic *Escherichia coli*, Biofilm

## Abstract

**Background:**

Increased resistance to antimicrobial agents is a characteristic of many bacteria growing in biofilms on for example indwelling urinary catheters or in intracellular bacterial reservoirs. Biofilm-related infections caused by multidrug-resistant bacteria, such as extended-spectrum β-lactamase (ESBL)-producing Enterobacteriaceae, are a major challenge. The aim of this study was to investigate if a carbon monoxide-releasing molecule (CORM-2) has antibacterial effects against ESBL-producing uropathogenic *E. coli* (UPEC) in the biofilm mode of growth and following colonization of host bladder epithelial cells.

**Results:**

The effect of CORM-2 was examined on bacteria grown within an established biofilm (biofilm formed for 24 h on plastic surface) by a live/dead viability staining assay. CORM-2 (500 μM) exposure for 24 h killed approximately 60 % of the ESBL-producing UPEC isolate. A non-ESBL-producing UPEC isolate and the *E. coli* K-12 strain TG1 were also sensitive to CORM-2 exposure when grown in biofilms. The antibacterial effect of CORM-2 on planktonic bacteria was reduced and delayed in the stationary growth phase compared to the exponential growth phase. In human bladder epithelial cell colonization experiments, CORM-2 exposure for 4 h significantly reduced the bacterial counts of an ESBL-producing UPEC isolate.

**Conclusion:**

This study shows that CORM-2 has antibacterial properties against multidrug-resistant UPEC under biofilm-like conditions and following host cell colonization, which motivate further studies of its therapeutic potential.

## Background

Biofilm is defined as bacteria enclosed in a self-produced polymeric matrix adherent to a biotic or abiotic surface [1]. *Escherichia coli* (*E. coli)* growing in biofilm display a development in several steps. Attachment is first mediated by flagella, type 1 fimbriae, curli and polysaccharides followed by early development of biofilm architecture and a maturation step [2]. Bacterial biofilms are more resistant to the effects of an antimicrobial agent than planktonic bacteria [3]. Slow growth rates, induction of biofilm-specific phenotypes and stress response activation as well as restricted antibiotic penetration by biofilm architecture have been proposed to explain the reduced susceptibility [3]. Biofilm formation on indwelling devices such as urinary catheters increases the risk of urinary tract infections (UTIs) and results in considerable antibiotic use [4]. Uropathogenic isolates of *E. coli* (UPEC) are the most frequent isolate in catheterized patients with UTI symptoms [5], and biofilm producing UPEC are also more frequent among strains causing UTI relapse [6]. Moreover, it has recently been recognized that UPEC may form biofilm-like structures on and inside bladder epithelial cells [7, 8]. If UPEC invade the urothelial cells they may rapidly replicate and subsequently aggregate into biofilm-like intracellular bacterial communities (IBC). In the late stage of IBC formation, the IBCs break open causing flux of bacteria from the superficial bladder facet cells into the bladder lumen allowing for invasion of other bladder cells [7, 9]. UPEC have also been shown to establish small clusters of more persistent intracellular reservoirs within the underlying basal epithelium [10]. Intracellular reservoirs of UPEC are believed to go undetected by standard urine culture, be protected from host defense mechanisms and persist despite antibiotic therapy [9]. Interestingly, emerging data suggest that UPEC persisting within intracellular reservoirs may have a role in development of recurrent and chronic UTIs [8, 9, 11]. Commonly used antibiotic treatments failed to eradicate UPEC growing internalized within bladder epithelial cells, by a proposed combination of biofilm formation, metabolically quiescent bacteria and bladder urothelial barriers [12, 13]. Thus, treatment of biofilm-related UTI is a challenge and particularly if caused by multidrug-resistant UPEC isolates.

Multidrug-resistant extended-spectrum β-lactamase (ESBL)-producing *E. coli* have disseminated worldwide and a main reason for mortality caused by these bacteria is inadequate initial antimicrobial therapy [14]. ESBL-producing Enterobacteriaceae spp. have genes that code for the ESBL enzyme and different ESBL enzyme variants (TEM, SHV, CTX-M) have been identified. CTX-M β-lactamases confer resistance to third generation cephalosporins, like cefotaxime, and often against other classes of antibiotics such as fluoroquinolones [15]. Dissemination of ESBL-producing bacteria occurs by horizontal transfer of plasmids (mainly CTX-M plasmids) or clonal spread [16]. The CTX-M-15 dominance around the world is due to dissemination of the virulent UPEC clone ST131 [17]. Studies indicate that the close contact between cells during biofilm conditions facilitates plasmid transfer by conjugation, a phenomenon that may increase the development of multidrug resistance in biofilm [18].

New treatment strategies for multidrug-resistant bacteria may include approaches with more penetrant antimicrobials with activity against non-growing bacteria or biofilm. Carbon monoxide (CO) is a small gaseous molecule with anti-inflammatory and antimicrobial properties that is able to penetrate cell membranes [19]. Metal carbonyl compounds, commonly known as CO-releasing molecules or CORMs, that release CO in a controlled manner have been developed for therapeutic applications [20]. Antibacterial effects of ruthenium-based carbonyls (CORM-2 and CORM-3) are reported in *E. coli* K-12 strains*, Staphylococcus aureus* and *Pseudomonas aeruginosa* [21–23]. CORM-3 has been shown to rapidly deliver CO to the intracellular part of the bacteria and to inhibit respiration by reacting with cytochrome *bd* and *bo*’ [22]. However, the activity of CORMs is not restricted to impairment of the aerobic respiratory chain, as these compounds are also effective in near-anaerobic conditions [21]. Another proposed mechanism for the antibacterial effect of CORM-2 is generation of intracellular reactive oxygen species that cause DNA damage and death [24]. Many genes related to biofilm formation were modified by CORM-2 as shown by whole-genome transcription profiling of a non-pathogenic *E. coli* K-12 strain [25]. We have recently shown that CORM-2 has bactericidal effects on planktonic multidrug-resistant ESBL-producing UPEC isolates [26], but it is not yet known if CORM-2 will be effective against ESBL-producing UPEC isolates in biofilms.

The aim of the present work was to investigate the antibacterial effects of CORM-2 on ESBL-producing UPEC in the biofilm mode of growth and following colonization of host bladder epithelial cells.

## Methods

### Bacterial isolates and strains

Four ESBL-producing UPEC isolates (designated ESBL1, 6, 7, 9) and four non-ESBL-producing UPEC isolates (designated UPEC 2, 3, 4, 5) were obtained from the Department of Microbiology at Örebro University hospital, Sweden. The UPEC isolates were recovered from urine of standard care patients with indwelling urinary catheters and symptoms of UTI. The identity of the patients was anonymized prior to further analysis of the bacterial isolates. Ethical approval was not required for this study as no analysis of human subjects, human material or human data were made. Antimicrobial susceptibility testing was performed using methods recommended by the Swedish Reference Group for Antibiotics (www.sls.se/RAF) and the subcommittee on methodology (NordicAST) (www.nordicast.org). The ESBL-producing isolates were characterized regarding CTX-M type as previously described [27]. The *E. coli* clone ST131 was detected using two ST131-specific *pabB* SNP assays by real-time PCR as previously described [28]. The CTX-M types, clonal group ST131 and the antibiotic susceptibility of the ESBL-producing isolates are shown in Table 1. The commensal *E. coli* K-12 strain TG1 carrying a F-conjugative plasmid that promotes biofilm formation [29] was used as a positive control strain for biofilm formation. TG1 and the commensal *E. coli* K-12 strain MG1655 were used from laboratory stocks.

**Table 1:** Characteristics of the clinical isolates

Isolate	CTX-M subgroup	CTX-M type	ST131 clone	Antibiotic resistance
ESBL 1	CTX-M -9	CTX-M-24	-	CTX, CAZ, CTB, CIP, GEN, MEL, TMP
ESBL 6	CTX-M -1	CTX-M-15	+	CTX, CAZ, CTB, CIP, TMP
ESBL 7	CTX-M -1	CTX-M-15	+	CTX, CAZ, CIP, MEL, TMP
ESBL 9	CTX-M -9	CTX-M-14	-	CTX, CAZ, CIP, MEL, TMP
UPEC 2^a^				
UPEC 3^a^				
UPEC 4^a^				TMP
UPEC 5^a^				CIP

Antibiotic resistance of ESBL-producing isolates (ESBL) and non-ESBL-producing UPEC isolates (UPEC) isolated from urine of patients with UTI. The CTX-M subgroup, CTX-M type and sequence type (ST) 131 are indicated for the ESBL-producing isolates

Abbreviations: cefotaxime (CTX), ceftazidime (CAZ), ceftibuten (CTB), ciprofloxacin (CIP), gentamicin (GEN), mecillinam (MEL), nitrofurantoin (NIT), trimethoprim (TMP)

^a^susceptibility tested for NIT, MEL, TMP, CTX, CIP

### Analysis of biofilm formation

Overnight cultures in Difco Luria-Bertani (LB) broth (Lennox, Becton Dickinson, Franklin Lakes, USA) were used to inoculate (at 0.1 %) fresh minimal salt (MS) medium [21] on agitation to an optical density (OD_620_) of approximately 0.05. The bacteria were seeded into 96-well plastic plates (Nunc C96 Microwell plate, Nunc A/S, Roskilde, Denmark) and after 6 h at 37 °C exposed to sub-inhibitory concentrations (100 or 250 μM) of CORM-2 ([Ru(CO)_3_Cl_2_]_2_ (Sigma-Aldrich, St. Louis, MO, USA) or left untreated. After an additional 18 h of incubation under static conditions at 37 °C, biofilm formation was quantified by the crystal violet method as previously described [25]. The absorbance at 540 nm was measured by spectrophotometer (Thermo Labsystems, Multiscan Ascent).

### Live/dead viability staining assay

Overnight cultures of biofilm producing strains were grown on eight-well chambered cover glasses (Lab-Tek, Rochester NY) for 24 h at 37 °C. Planktonic bacteria were removed by gentle washing and the remaining biofilm was exposed to CORM-2 (500 μM), cefotaxime (0.512 μg/ml (Sigma-Aldrich), corresponding to 4 x MIC for UPEC isolate 2) or vehicle (DMSO 2.5 % or sterile water) for 24 h at 37 °C. MIC for CORM-2 has previously been reported to be 500 μM in *E. coli* [21]. Thereafter, the biofilms were washed with 0.85 % NaCl and incubated with a live/dead viability staining assay (BacLight, Life Technologies, Leiden, The Netherlands) according to kit instructions. Biofilm images were obtained using a Leica TCS confocal laser scanning microscope (Leica Microsystems, Heidelberg GmbH, Wetzlar, Germany). The ratio of dead bacteria (expressed as a percentage) was calculated by manual counting of four randomly selected quadrants (in total covering ¼ from each image) from a representative part of each well.

### Bacterial viability under planktonic growth conditions

Overnight LB-cultures were used to inoculate (at 0.1 %) fresh MS-medium. In experiments representing the exponential growth phase, cultures were grown to an OD_620_ of approximately 0.1 to reach early exponential phase. The bacterial concentration of the initial inoculums used in these experiments was approximately 10^7^-10^8^ CFU/ml. In experiments representing the stationary growth phase, cultures were grown with aeration at 37 °C for approximately 14 h. The bacterial concentration of the initial inoculums used in these experiments was approximately10^9^ CFU/ml. The bacteria were treated with CORM-2 (500 μM), DMSO (controls) or cefotaxime (0.512 μg/ml) for 4 and 24 h. After treatment, the bacterial viability was determined by plating serial dilution on TSA plates followed by counting the CFU numbers on overnight cultures at 37 °C. Bacterial CFU/ml was determined by using the mean from two dilutions.

### Human bladder epithelial cells

The human bladder epithelial cell line 5637 (ATCC HTB-9; American Type Culture Collection Manassas, USA) was grown in Dulbecco’s Modified Eagle medium (Sigma-Aldrich) supplemented with 10 % fetal bovine serum, 2 mM L-glutamine, 1 mM non-essential amino acids, 100 U/ml penicillin and 100 μg/ml streptomycin (all from Invitrogen, Paisley, UK) in a humidified incubator at 37 °C with 5 % CO_2_.

### Bacterial viability following colonization of host bladder cells

Overnight LB-cultures of ESBL-producing isolate 6, 7 and 9 were grown at 37 °C in static LB-broth to facilitate induction of type-1 fimbriae expression [30]. The 5637 bladder epithelial cells were grown in 24-wells until confluent and infected with bacteria in DMEM supplemented with 2 % fetal bovine serum, 2 mM L-glutamine and 1 mM non-essential amino acids. A multiplicity of infection (MOI) of approximately 20 bacteria per host cells was used. The 24 well plates were centrifuged at 600 x g for 10 min to facilitate the attachment of bacteria to epithelial cells followed by incubation for 2 h. A gentamycin protection assay was used to support intracellular growth of UPEC as previously described [12]. Briefly, cells were incubated with 100 μg/ml of gentamycin in DMEM for 2 h to kill extracellular bacteria. Following washes with PBS, the cells were incubated with DMEM containing a submaximal concentration of gentamycin (10 μg/ml) for another 14 h to limit potential leak of gentamycin into the bladder cells. Cell layers were washed and exposed for 4 h to CORM-2 (250 and 500 μM), cefotaxime (0.512 μg/ml) or controls (DMSO or Ru(DMSO)_4_Cl_2_). Ru(DMSO)_4_Cl_2_ (Strem chemicals Inc, Newburyport, MA, USA) is a negative control for CORM-2 where the CO groups have been replaced by DMSO. The host cell viability and adherence were checked throughout the experiments. After incubation, the cells were washed, lysed with 0.5 % Triton X-100 and serially diluted and counted by plating on TSA agar plates as described before.

### Immunofluorescence of UPEC in the presence of host bladder epithelial cells

Bladder 5637 cells were seeded onto well glass chamber slides (SPL Lifesciences Co., Ltd., Pocheon-city, Gyeonggi-Do, Korea), grown to subconfluency and infected with ESBL isolates as described above. After the incubation with gentamycin (totally 16 h), the cells were washed with PBS and fixed for 10 min in 4 % paraformaldehyde. Cells were washed and incubated with 1 % bovine serum albumine (BSA) to block unspecific binding of the antibodies. Extracellular bacteria were labelled by incubation with a goat polyclonal anti-*E. coli* antibody (Abcam, Cambridge, UK) diluted 1:500 in PBS with 1 % BSA for 60 min at room temperature (RT), washed and then incubated with a secondary donkey anti*-*goat IgG-DyLight549 conjugated antibody (Jackson ImmunoResearch Europe Ltd., Suffolk, UK) (red fluorescence) diluted 1:500 (in PBS with 1 % BSA) for 90 min at RT. In order to label intracellular bacteria the host cells were permeabilized with 0.5 % Triton X-100 for 10 min, washed and reprobed with the goat anti-*E. coli* antibody for 60 min. After wash, the bacteria were stained with a secondary rabbit anti-goat IgG-FITC conjugated antibody (Sigma-Aldrich) (green fluorescence) diluted 1:400 (in PBS with 1 % BSA) for 100 min at RT. Samples were mounted with Vectashield Mounting media (Vector Laboratories Inc., Burlingame, CA, USA) containing 4′, 6-diamidino-2-phenylindole (DAPI) for staining of nuclei. Bacteria that stained both red and green were scored as adherent extracellular bacteria, while those that stained only green were scored as intracellular bacteria. Images were obtained and processed using an Olympus BX60 fluorescence microscope equipped with an Olympus DP71 camera and Adobe Photoshop software.

### Statistical analysis

Data are expressed as mean ± SEM. Differences between two groups were assessed by the unpaired two-tailed Students *t*-test or by the one sample *t*-test when controls were normalized to 1. One-way ANOVA followed by Bonferroni test was used for multiple comparisons. Results were considered statistically significant at *p* < 0.05. n = number of independent experiments.

## Results

### Effect of CORM-2 on biofilm formation

**Fig. 1:**
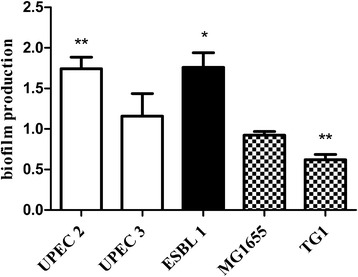
The antibacterial effect of CORM-2 on biofilm formation. Effect of CORM-2 (250 μM) on biofilm formation in non-ESBL-producing UPEC isolates (UPEC 2 and 3), in an ESBL-producing isolate (ESBL1) and in the non-pathogenic *E. coli* K-12 strains MG1655 and TG1. Biofilm formation was measured by the crystal violet method 18 h after exposure to CORM-2 and expressed as relative changes compared to untreated controls. The data are presented as mean ± SEM from at least three independent experiments. *P < 0.05, **P < 0.01, CORM-2 versus control

A detectable basal biofilm formation (A_540_> 0.1) was found in ESBL-producing isolate 1, UPEC isolates 2 and 3 and the non-pathogenic *E. coli* K-12 strains MG1655 and TG1 (Table 2). Only these isolates were used for further biofilm studies. A sub-inhibitory concentration of CORM-2 (250 μM) significantly increased biofilm production in ESBL-producing isolate 1 (*p* = 0.017) and in UPEC isolate 2 (*p* = 0.0058) (Fig. 1). A lower concentration of CORM-2 (100 μM) had only minor effects on biofilm formation (data not shown). TG1, a well-established biofilm-producing *E. coli* K-12 strain, showed reduced biofilm formation (*p* = 0.0045) in response to CORM-2. Biofilm formation in UPEC isolates 3 and the K-12 strain MG1655 was not affected by CORM-2 (Fig. 1).

**Table 2:** The basal biofilm production of the different isolates

A_540_ < 0.1	A_540_ 0.1-1	A_540_ > 1
ESBL 6, ESBL 7, ESBL 9, UPEC 4, UPEC 5	ESBL 1, UPEC 2, UPEC 3, MG1655	TG1

Basal biofilm production after 24 h in ESBL-producing isolates (ESBL), non-ESBL-producing UPEC isolates (UPEC) and non-pathogenic *E. coli* K-12 strains (MG1655 and TG1) evaluated by the crystal violet method and measured by absorbance levels (A_540_). Detectable biofilm production was considered at A_540_ ≥ 0.1. Mean values from three to six independent experiments

### Effect of CORM-2 on bacterial viability within an established biofilm

**Fig. 2:**
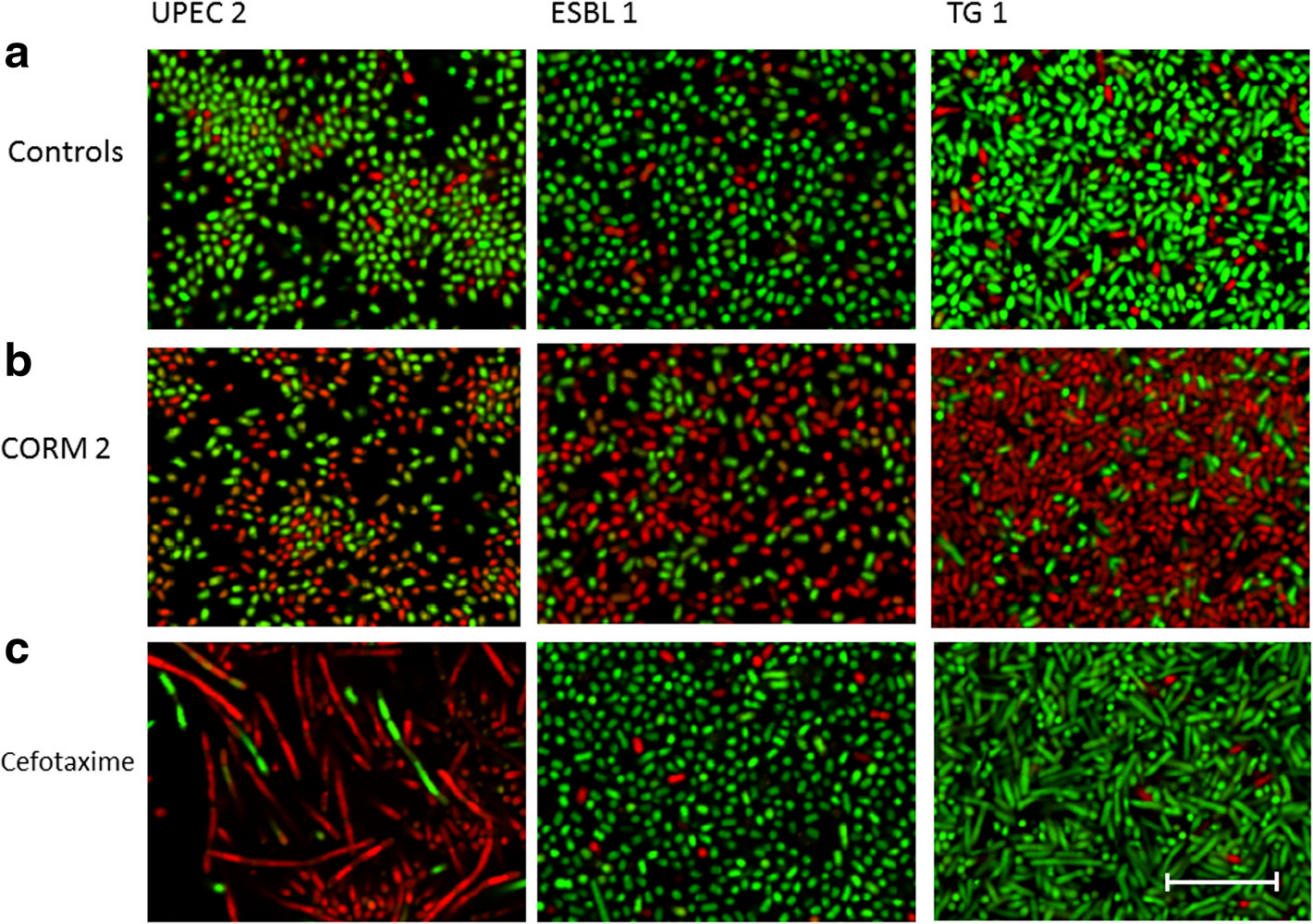
Visualisation of the antibacterial effect of CORM-2 and cefotaxime on established biofilm. Effect of CORM-2 and cefotaxime on bacterial viability within an established biofilm (biofilm grown for 24 h) evaluated by a live/dead viability staining assay using confocal microscopy. Live bacteria with intact cell membrane are stained green (SYTO9) and dead bacteria with damaged cell membrane are stained red (propidium iodine). Photographs show representative areas from the chamber slides; from left to right isolate UPEC isolate 2, ESBL isolate 1 and K-12 strain TG1 and from top-down **a** controls, **b** 24 h CORM-2 (500 μM), **c** 24 h cefotaxime (0.512 μg/ml). Scale bar = 10 μm. Representative photographs from three independent experiments are shown

We next examined the effect of CORM-2 (500 μM) on UPEC isolate 2, ESBL-producing isolate 1 and K-12 strain TG1 when grown within an established biofilm (biofilm formed for 24 h on plastic surface). To quantify the effects of CORM-2 on bacterial viability a live/dead viability staining assay and confocal microscopy were used. Pilot experiments showed that 4 h of exposure to CORM-2 had minor effect on viability but that the viability of all isolates was clearly reduced by CORM-2 (500 μM) after 24 h of exposure (Fig. 2). The percentage dead bacteria was 60 ± 16, 61 ± 18 and 85 ± 9 for ESBL isolate 1, UPEC isolate 2 and strain TG1, respectively (Fig. 2, Table 3). Cefotaxime (0.512 μg/ml), a hydrophilic drug with low permeable [31, 32], killed UPEC isolate 2 (76 ± 8 %) and long filamentous bacteria were observed by confocal microscopy (Fig. 2). However, no effect on viability was noted by cefotaxime in ESBL-producing isolate 1 or the K-12 strain TG1 (Fig. 2, Table 3). The number of dead bacteria in DMSO-treated controls was low (Fig. 2, Table 3). The CO-free molecule Ru(DMSO)_4_Cl_2_, used as a negative control for CORM-2, had similar effects as DMSO on viability (data not shown).

**Table 3:** Quantitative data from the live/dead viability assay

Stimuli	Mean % ± SEM	*P value*
UPEC 2 control	5 ± 2	
UPEC 2 CORM-2	61 ± 18	0.0208*
UPEC 2 cefotaxime	76 ± 8	0.0011**
ESBL 1 control	6 ± 2	
ESBL 1 CORM-2	60 ± 16	0.0135*
ESBL 1 cefotaxime	5 ± 1	
TG1 control	13 ± 2	
TG1 CORM-2	85 ± 9	0.0003***
TG1 cefotaxime	4 ± 1	

Data show the ratio of dead bacteria (expressed as a percentage) after 24 h exposure to CORM-2 (500 μM), cefotaxime (0.512 μg/ml) or control (DMSO) evaluated by a live/dead viability assay. The ratio of dead bacteria was calculated by manual counting of four randomly selected quadrants (in total covering ¼ from each image) from a representative part of each well. The data are presented as mean ± SEM. (*n* = 3) *P < 0.05, **P < 0.01, ***P <0.001; CORM-2 or cefotaxime versus control

### Effect of CORM-2 on planktonic bacteria in different growth phases

**Fig. 3:**
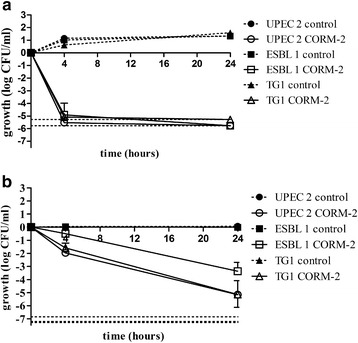
The antibacterial effect of CORM-2 in the exponential and stationary growth phase. A comparison of the effect of CORM-2 exposure in the exponential growth phase and in the stationary phase in ESBL isolate 1, UPEC isolate 2 and K-12 strain TG1. **a** Cultures were grown to early log phase in MS-broth and then exposed to CORM-2 (500 μM) or DMSO (control). **b** Cultures were grown to stationary phase in MS-broth and then exposed to CORM-2 (500 μM) or DMSO (control). Growth was calculated as the numbers of CFU/ml in treated cultures or controls divided by the number of CFU/ml formed upon the plating of the initial inoculums and expressed as log CFU/ml. The data are presented as mean ± SEM from at least three independent experiments. Lower grids show the detection limit

The antibacterial effect of CORM-2 was compared in planktonic cultures (ESBL-producing isolate 1, UPEC isolate 2 and strain TG1) when exposed in the stationary growth phase or in the exponential growth phase. Unexposed controls in the exponential growth phase showed an increased growth of ~ 1.5 log units during the 24 h experiment (Fig. 3a). CORM-2 (500 μM) evoked a fast bactericidal effect, in all isolates, with a reduction of bacterial counts by > 3 log units within 4 h (Fig. 3a). Unexposed controls in the stationary growth phase showed no increased growth during the 24 h experiment (Fig. 3b). CORM-2 demonstrated a delayed inhibitory response in the stationary growth phase, but a bactericidal effect was found after 24 h (Fig. 3b). Separate experiments showed that colonies of ESBL-producing isolate 1 that survived CORM-2 treatment in the stationary phase, was effectively killed when re-exposed in the exponential growth phase. Planktonic cultures of UPEC isolate 2 and the K-12 strain TG1 were susceptible to cefotaxime with a reduction of growth by 0.32 ± 0.047 and 1.5 ± 0.47 log units, respectively. ESBL-producing isolate 1 was resistant to cefotaxime.

### Effect of CORM-2 following colonization of host bladder epithelial cells

**Fig. 4:**
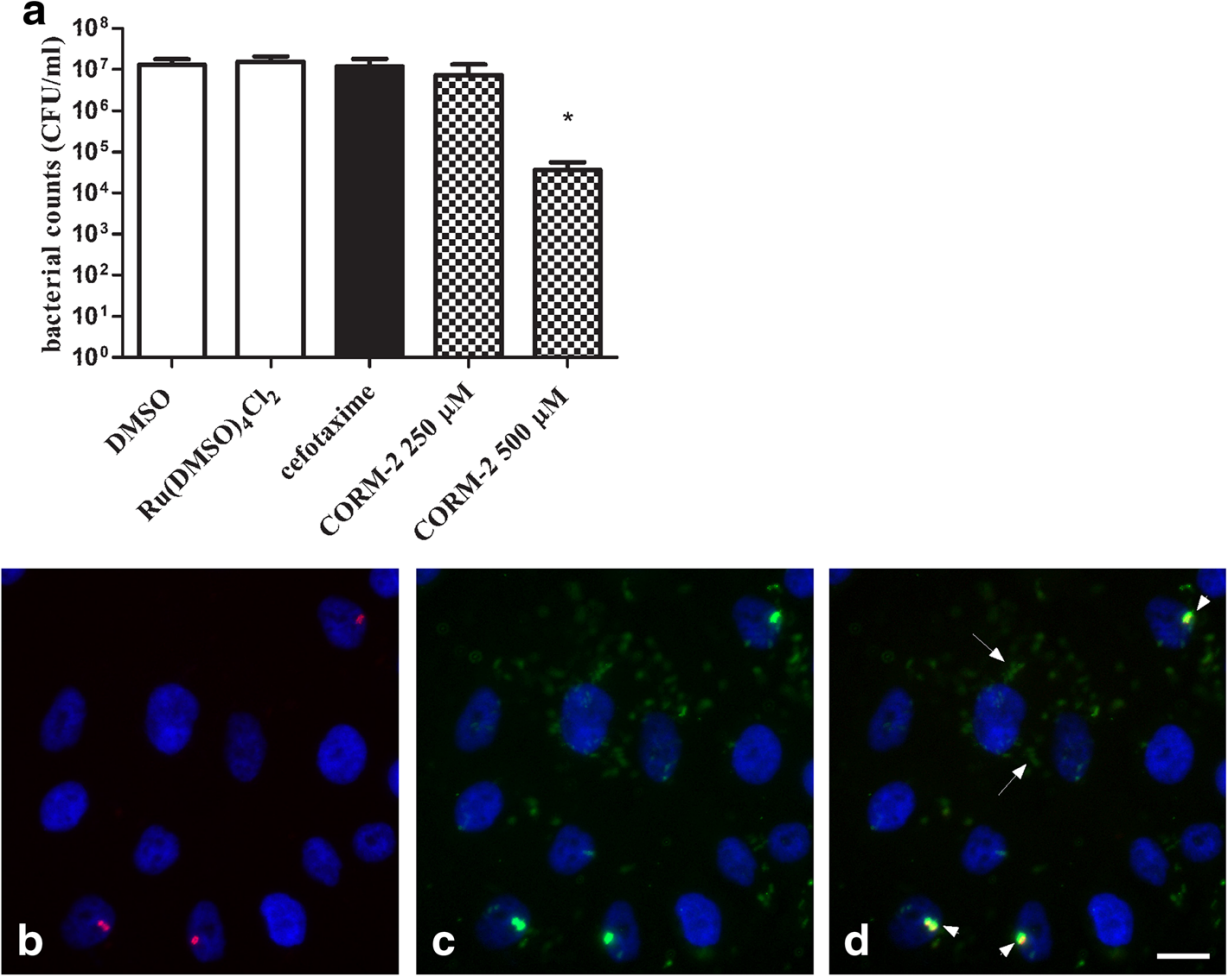
The antibacterial effect of CORM-2 on colonization of host cells and localization of colonized bacteria. Quantification and immunofluorescence staining of ESBL-producing isolate 6 following colonization of 5637 bladder epithelial cells. A gentamycin protection assay was used to support intracellular growth of the bacteria. **a** Host bladder 5637 cells were lysed and the lysate serially diluted and plated on TSA plates for quantification of bacterial count (CFU/ml). Bacterial counts in cell lysate was evaluated after exposure for 4 h to DMSO, the CO-free molecule Ru(DMSO)_4_Cl_2_ (500 μM), cefotaxime (0.512 μg/ml) or CORM-2 (250 and 500 μM). Data are presented as mean ± SEM from four independent experiments. *P < 0.05, CORM-2 versus DMSO. **b**-**d** Immunofluorescence staining of ESBL-producing isolate 6 following infection of 5637 bladder epithelial cells. Staining of the bladder cell nuclei was performed with DAPI and is shown in blue. ESBL isolate 6 are stained **b**) in red (extracellular) prior to permeabilization, and **c**) in green (extracellular and intracellular) after permeabilization. **d** Merged image of B and C where several intracellular bacteria are seen as green stain (arrows) and extracellular bacteria are shown as merged red and green (yellow) stain (arrowhead). Scale bar = 10 μm

Separate experiments demonstrated that ESBL-producing isolates 7 and 9 showed weak colonization of human 5637 bladder epithelial cells, while ESBL isolate 6 showed a consistent ability to colonize host bladder cells. ESBL isolate 1 was excluded in the colonization experiments due to its gentamycin resistant profile. Infected 5637 bladder cells were less confluent than uninfected 5637 cells and approximately 50 % of the cells were viable after 16 h of infection. Exposure to CORM-2 at 500 μM, but not 250 μM, for 4 h significantly (*p* = 0.0235) reduced the bacterial counts of ESBL isolate 6 in the presence of 5637 bladder cells by approximately 2.5 log units (Fig. 4a). A negative control for CORM-2, Ru(DMSO)_4_Cl_2_, and cefotaxime did not reduce the bladder cell colonization of ESBL isolate 6 (Fig. 4a). CORM-2 did not affect 5637 bladder cell confluence or adherence during the 4 h exposure time. A double immunofluorescence staining procedure was performed to investigate the localization of colonized bacteria. Bacteria were found both attached to host bladder cells in the extracellular space and also intracellular (Fig. 4b-d).

## Discussion

Several recent publications highlight the urgent need for new therapies against multidrug-resistant *E. coli* [33, 34]. Multidrug-resistant UPEC associated with biofilm on urinary catheters and within bladder reservoirs are particularly problematic to eradicate due to a combination of antibiotic resistance and antibiotic penetration barriers. In the present study, the overall biofilm formation on plastic abiotic surface was low in the UPEC isolates compared to the positive control *E. coli* K-12 strain TG1. A significant variation in the capacity of different *E. coli* isolates to form biofilm exists and both genetic and environmental factors affect the biofilm phenotype in a complex manner [35]. Growth medium composition has significant impact on biofilm formation [35] and some virulence-associated genes appear to be more common in *E. coli* strains with strong biofilm production [36]. A flow chamber biofilm model system would possibly have improved the biofilm formation of the UPEC isolates in the present study. When UPEC isolates with detectable biofilm formation were exposed to CORM-2 an increased biofilm formation was found in two out of three isolates, suggesting that increased biofilm production may be a defence mechanism against CORM-2. A previous study performed in an *E. coli* K-12 strain showed that CORM-2 increased transcription of several biofilm-related genes and increased biofilm production when grown in LB-broth [25]. The two non-pathogenic K-12 strains used in our study were unaffected or showed a reduced biofilm formation when exposed to CORM-2. Biofilm formation was examined in a concentration (250 μM) that is sub-inhibitory for UPEC [26], however the growth of planktonic TG1 was reduced at this concentration (data not shown), which may explain the impaired ability to form biofilm.

CORMs represent a novel class of antimicrobials and are so far non-explored agents in the field of eradication of biofilm growth caused by UPEC. The CO-releasing profile of CORMs has recently been re-evaluated [20]. It has been proposed that CORMs are internalized into bacteria by a Trojan horse mechanism, rather than that CO diffuses into the cell [22, 37]. CORM-2 was able to reduce the bacterial viability of ESBL-producing and non-ESBL-producing UPEC isolates, as well as the K12 strain TG1, when grown within an established biofilm. Thus, CORM-2 seems able to penetrate biofilm-like architecture and kill bacteria in biofilms as confirmed by the live/dead viability assay and confocal microscopy.

The ESBL-producing isolate was resistant to the cephalosporine antibiotic cefotaxime and, as expected, cefotaxime did not affect the viability of the ESBL isolate within an established biofilm. However, exposure to cefotaxime reduced the viability of the non-ESBL producing UPEC isolate and long filamentous bacteria were visualized by confocal microscopy. Filamentous bacteria can appear as a consequence of bacterial stress, including exposure to beta-lactam antibiotics that through inhibition of penicillin-binding protein-3 in the peptidoglycan layer prevents septa formation and cell division [38]. *E. coli* strain TG1, with the highest biofilm production, was effectively killed (85 %) by CORM-2 but was insensitive to cefotaxime. Cefotaxime is mainly active against dividing bacteria and has low efficiency for adherent bacteria within a biofilm matrix compared to planktonic cultures [39]. Our data confirmed that planktonic TG1 cultures, but not bacterial biofilms, were sensitive to cefotaxime. A limitation of this study is that the biofilm formation by the clinical UPEC isolates was low, and that the effects of CORM-2 on biofilms may have been overestimated. However, CORM-2 was effective against TG-1 biofilms suggesting that CORM-2 is able to penetrate and reach targets within more mature biofilms. In agreement with our findings, CORM-2 was demonstrated to kill a majority of clinical isolates of *P. aeruginosa* within an established biofilm [40]. The main mechanism by which CORM-2 inhibited *P. aeruginosa* biofilm appeared to be interference with the respiratory chain rather than production of reactive oxygen species [40].

Impaired penetration of antibiotics is not the only mechanism associated with biofilm-related antibiotic resistance. Another characteristic of a biofilm is the presence of a large subpopulation of bacteria in a dormant persister state [38]. Bacteria in a dormant state are found in biofilms and in stationary phase cultures, but not in cultures in the exponential growth phase. Bacteria that have entered a dormant state are transiently tolerant to antibiotics by mechanism such as reduced translation and cell wall synthesis [41, 42]. The effects of CORM-2 in the exponential and stationary growth phase were investigated to reveal if slower growing bacteria are more resistant to CORM-2. CORM-2 reduced bacterial viability in the stationary phase, but the inhibition was markedly delayed compared to cultures in the exponential phase demonstrating that metabolically active bacteria are more susceptible to CORM-2. Bacteria that survived the CORM-2 treatment in stationary phase experiments were killed when re-exposed to CORM-2 in the exponential phase, which suggest that stationary phase bacteria may represent dormant persisters with a transient tolerance to CORM-2. The higher cell density in the stationary phase may in part explain the delayed efficacy of CORM-2 in the stationary phase since the effective drug concentration for each bacterium may be lower.

The 5637 human bladder epithelial cell line was infected with an ESBL-producing isolate and the gentamycin protection assay was used to support intracellular localization. There are known difficulties in replicating the conditions required for formation of intracellular biofilm-like bacterial communities in vitro*.* However, UPEC have been shown to invade 5637 bladder epithelial cells but they rarely form large IBC inclusions as found in superficial bladder facet cells in vivo [11, 43]. The immunofluorescence method used in the present study, which is able to distinguish between extra- and intracellular bacteria, demonstrated that the ESBL isolate could adhere to and invade 5637 bladder epithelial cells. In agreement with previous studies [11, 43, 44], the intracellular bacteria were dispersed in the cytosol and not packed in biofilm-like aggregates. Exposure to CORM-2 significantly reduced the bacterial counts following bladder epithelial cell colonization. The CO-free molecule Ru(DMSO)_4_Cl_2_ elicited no reduced viability, verifying that CO is required for the antibacterial effect of CORM-2. The cell cytotoxicity of CORM-2 (100-500 μM) has previously been observed to be low in 5637 bladder cells and CORM-2 even seemed to have a cytoprotective effect after 4 h [26]. The exposure time to CORM-2 was therefore restricted to 4 h in our experiments and no detrimental effects on the cells were observed. It may be argued that cytotoxic effects of CORM-2 on host bladder cells may enhance its efficacy against internalized UPEC by disrupting cell barriers. However, no correlation between 5637 bladder cell cytotoxicity and killing of intracellular UPEC by several antibiotics was found using a similar cell-culture based assay as in our study [12]. Nevertheless, we cannot exclude that some effects of CORM-2 on the host bladder cells or on bacterial efflux mechanisms from the 5637 cells contribute to the antibacterial effects of CORM-2 on colonized bacteria. Taken together, CORM-2 has an interesting antibacterial profile by being effective against multidrug-resistant UPEC protected in biofilms and in intracellular reservoirs. It will be of interest to examine whether CORMs alone or in combination with currently available antibiotics will have antibacterial effect on intracellular and persistent UPEC in mouse UTI models. The search of CORMs with suitable properties for the delivery of CO and with low cytotoxicity is an ongoing and active area of research. New CORMs with desirable therapeutic profiles and clinical compatibility are appearing, but more fundamental knowledge on the chemistry, cellular targets and molecular biology of CORMs is needed before these compounds can be used clinically as antimicrobial agents [20, 45, 46].

The ESBL-producing isolates 6 and 7 belong to the *E. coli* ST131 clone that currently represents one of the most dominant groups of multidrug-resistant *E. coli* globally [47]. The ST131 clone, which produces CTX-15, is thought to be successful due to a combination of antibiotic resistance and virulence. Here, we show that ESBL isolate 6 was able to adhere to and invade host bladder epithelial cells, suggesting that this isolate has a capacity to form intracellular reservoirs and persist in the bladder. In agreement, a ST131 reference strain *E. coli* EC958 has previously been demonstrated to invade bladder epithelial cells both in mouse studies [48] and in vitro studies [49].

## Conclusions

This study demonstrates that CORM-2 is able to reduce the bacterial viability of multidrug-resistant UPEC in biofilm-like conditions and following colonization of human bladder epithelial cells. CORMs will be interesting candidate drugs to investigate further in studies of recurrent and chronic UTI caused by multidrug-resistant UPEC.

### Ethics approval and consent to participate

Not applicable.

### Consent for publication

Not applicable.

### Availability of data and materials

All the data supporting the findings is contained within the manuscript or will be shared upon request.

## Abbreviations

BSA: Bovine serum albumin
CAZ: Ceftazidime
CIP: Ciprofloxacin
CO: Carbon monoxide
CORM-2: Carbon monoxide-releasing molecule-2
CORM-3: Carbon monoxide-releasing molecule-3
CTB: Ceftibuten
CTX: Cefotaxime
DAPI: 4′, 6-diamidino-2-phenylindole
*E. coli*: *Escherichia coli*
ESBL: Extended-spectrum β-lactamase
GEN: Gentamicin
IBC: Intracellular bacterial communities
LB: Luria-Bertani broth
MEL: Mecillinam
MOI: Multiplicity of infection
MS: Minimal salt medium
NIT: Nitrofurantoin
*P. aeruginosa*: *Pseudomonas aeruginosa*
TMP: Trimethoprim
UPEC: Uropathogenic *E. coli*
UTI: Urinary tract infection
RT: Room temperature
